# MicroRNA Profile of Patients with Chronic Limb-Threatening Ischemia

**DOI:** 10.3390/diagnostics10040230

**Published:** 2020-04-17

**Authors:** Muzammil H. Syed, Abdelrahman Zamzam, Jason Valencia, Hamzah Khan, Shubha Jain, Krishna K. Singh, Rawand Abdin, Mohammad Qadura

**Affiliations:** 1 Division of Vascular Surgery, St. Michael’s Hospital, Toronto, ON M5B 1W8, Canada; muzammil.syed@mail.utoronto.ca (M.H.S.); abdelrahman.zamzam@unityhealth.to (A.Z.); jvalenci@uoguelph.ca (J.V.); hamzah.khan@mail.utoronto.ca (H.K.); jains@ucalgary.ca (S.J.); 2 Department of Medical Biophysics, Schulich School of Medicine and Dentistry, University of Western Ontario, London, ON N6A 5C1, Canada; krishna.singh@uwo.ca; 3 Department of Medicine, McMaster University, Hamilton, ON L8S 4K1, Canada; rawand.abdin@medportal.ca; 4 Keenan Research Centre for Biomedical Science, Li Ka Shing Knowledge Institute of St. Michael’s Hospital, Toronto, ON M5B 1W8, Canada; 5 Department of Surgery, University of Toronto, Toronto, ON M5T 1P5, Canada

**Keywords:** chronic limb threatening ischemia, microRNA, next generation sequencing, quantitative polymerase chain reaction

## Abstract

Chronic limb-threatening ischemia (CLTI) results in devastating complications such as lower-limb amputations. In this study, a genome-wide plasma microRNAs (miRNA) sequencing was performed to identify miRNA(s) associated with CLTI. Blood samples were collected from early stage CLTI patients (ABI < 0.9) and non-PAD controls (ABI ≥ 0.9) for 3 experiments: discovery phase (*n* = 23), confirmatory phase (*n* = 52) and validation phase (*n* = 20). In the discovery phase, next generation sequencing (NGS) was used to identify miRNA circulating in the plasma CLTI (*n* = 13) patients, compared to non-PAD controls (*n* = 10). Two down-regulated miRNAs (miRNA-6843-3p and miRNA-6766-5p) and three upregulated miRNAs (miRNA-1827, miRNA-320 and miRNA-98-3p) were identified (≥2-fold change). In the confirmatory phase, these 5 deregulated miRNAs were further investigated in non-PAD (*n* = 21) and CTLI (*n* = 31) patients using qRT-PCR. Only miRNA-1827 was found to be significantly upregulated (≥3-fold, *p*-value < 0. 001) in the CLTI group. Lastly, to minimize the influence of confounding factors, miRNA-1827 plasma levels were validated in a third cohort of CLTI patients (*n* = 10) matched to non-PAD controls (*n* = 10). Our analysis demonstrated that miRNA-1827 expression was increased in the CLTI cohort (≥2-folds, *p*-value < 0.001). In summary, circulating miRNA-1827 is significantly elevated in patients with CLTI.

## 1. Introduction

Peripheral arterial disease (PAD) is a devastating disease in which atherosclerotic plaque buildup leads to a narrowing of peripheral arteries in the lower limb, manifesting as ischemic pain [1,2]. PAD affects more than 200 million patients worldwide, with 12 million people suffering from the disease in North America alone [3]. Common PAD symptoms include pain induced by walking which resolves at rest (claudication). 

Moreover, PAD can progress in disease state and severity to chronic limb threatening ischemia (CLTI) [4]. CLTI manifests as rest and/or night pain (early stages), or non-healing ulceration with tissue loss and gangrene (late stages). Patients with early CLTI often require arterial reconstructive surgeries to improve limb perfusion, whereas patients with late CLTI often require major limb amputation [5,6]. Studies suggest that within 2 years of diagnosis, CTLI patients have 25% mortality rate as well as 25% amputation rate [7]. Mortality after limb amputations is even higher, with studies suggesting a 30% mortality rate within 2 years [8]. 

Several clinical methods exist to identify the presence of CLTI, such as ankle brachial index (ABI), color doppler ultrasound examination and transcutaneous oxygen pressure [9,10,11]. However, little information exists regarding blood-based biomarkers of CTLI. Research into circulating biomarkers for CLTI would shed considerable light on the disease, and significantly contribute to the discovery of new and innovative diagnostic tools. 

MicroRNAs (miRNA) have gained significant traction and popularity as potential novel indicators of cardiovascular disease progression [12]. They are short (approximately 22 nucleotides), endogenous, non-coding, single stranded RNA that can post-transcriptionally modulate gene expression through attenuation of mRNA stability [13,14]. Furthermore, studies have shown that expression of specific miRNAs can reliably be utilized as robust biomarkers for specific diseases. Therefore, the purpose of this study was to investigate the miRNA plasma profile of patients with CLTI in order to discover potential diagnostic biomarkers for this disease. In this study, next generation genome-wide sequencing and real-time quantitative polymerase chain reaction (RT-qPCR) were utilized to investigate the plasma miRNA profile of CLTI and non-PAD patients [15,16,17].

## 2. Materials and Methods 

### 2.1. Ethics Approval

This study was approved by The Unity Health Toronto Research Ethics Board at St. Michael’s Hospital—University of Toronto in Ontario (#16-375, 8 February 2017), Canada. Informed consent was obtained from all participants. 

### 2.2. Patient Selection 

This pilot study was divided into a series of three experimental phases: discovery, confirmation and validation phases. For all experiments, non-PAD and CLTI patients referred to vascular surgery ambulatory clinics or emergency department at St. Michael’s Hospital were considered and recruited for this study. The non-PAD cohort was defined as patients with a normal arterial US of the lower limbs, ankle brachial index (ABI) of ≥ 0.9 and/or toe brachial index (TBI) of ≥ 0.67, palpable distal pulses, and no significant clinical history of claudication. Patients with early stage CLTI were defined as ABI < 0.9 or TBI < 0.67 with a resting ankle pressure (AP) < 40 mm Hg or toe pressure (TP) < 30 mm Hg, as per the Rutherford classification criteria (stage 4). The following patients were excluded: 1) patients on chemotherapy or biological anti-inflammatory agents, 2) patients with renal disease (stages 4 and 5) as per the Kidney Disease Outcomes Quality Initiative clinical guidelines and, 3) patients with acute limb ischemia or a diagnosis of malignancy. 

### 2.3. Baseline Measurements 

Type II Diabetes Mellitus (DM) was defined as glycosylated hemoglobin A1c ≥ 6.5% or the use of anti-diabetic medication. Hyperlipidemia was defined as total cholesterol > 5.2 mmol/L or triglyceride > 1.7 mmol/L or the use of anti-hyperlipidemic medication. Hypertension was defined as systolic blood pressure ≥ 130mm Hg or diastolic pressure ≥ 80 mm Hg or use of antihypertensive medication. Renal disease was defined as estimated glomerular filtration rate less than 30 mL/min/1.73 m^2^, as per the Kidney Disease Outcomes Quality Initiative 2002 guidelines. Prior history of stroke or transient ischemic attack (TIA) was recorded for each patient, as well as smoking status.

### 2.4. PAD Assessment and Sample Processing

In all subjects, a thorough physical exam and complete medical history was obtained from each patient. Each patient received lower limb arterial ultrasound (US) including ABI or TBI were recorded for each patient. Blood samples were drawn into vacutainer tubes containing EDTA. Plasma was then extracted from this blood via centrifugation at 3000 rpm for 10 min 4 °C. 

### 2.5. miRNA Isolation, Quantification and Purity Analysis

Total RNA was extracted from the plasma samples using TRIzol (ThermoFisher, Waltham, MA, USA). Each sample was purified using miRNAeasy mini kit (Qiagen, Germantown, MD, USA) according to manufacturer’s instructions. RNA quality was measured using a Nanodrop spectrophotometer (ND-1000, Nanodrop Technologies, Waltham, MA, USA). RNA integrity was determined by gel electrophoresis. miRNA concentrations were measured by a Qubit RNA HS Assay on a Qubit fluorometer (ThermoFisher, Waltham, MA, USA). A small RNA library preparation was performed following the NEB NEBNext Multiplex Small RNA Library Prep Set for Illumina protocol small by The Centre for Applied Genomics at The Hospital for Sick Children (Toronto, ON, Canada). RNA libraries were quantified by qPCR using the Kapa Library Quantification Illumina/ABI Prism Kit protocol (KAPA Biosystems, Boston, MA, USA). Libraries were pooled in equimolar quantities and single-end sequenced on Rapid Run Mode flow cell with the V3 sequencing chemistry on an Illumina HiSeq 2500 platform following Illumina’s recommended protocol to generate single-end reads of 50-bases in length.

### 2.6. miRNA Bioinformatics Analysis 

miRNA bioinformatics data analysis was carried out by The Centre for Applied Genomics at The Hospital for Sick Children. Raw reads were preprocessed for adaptor trimming, quality and size selection using Trim Galore v0.2.8 (Babraham Bioinformatics, Cambridge, UK). The quality of the data was assessed using FastQC v0.11.2. For each sample, raw reads were preprocessed for adapter and quality trimming using Trim galore! (v0.4.1, Babraham Institute, Cambridge, UK). Adapter trimming was performed with stringency 5, low-quality ends were trimmed if they were below the minimum Phred (quality score) of 20nt (nucleotides). A minimum of 17 nt was the length cut-off as reads were shortened after the trimming. In terms of reads longer than 26 nt, these reads were trimmed using the FASTX toolkit v0.6.1. The quality of the trimmed reads was re-assessed with FASTX toolkit v0.6.1. The obtained aligned reads were classified as miRNA, RefSeq genes, RefSeq coding regions and rRNA using BEDtools v2.14.2. The miRNA counts in each sample, differential expression analysis was performed using the DESEq v1.10.1 Bioconductor package within R, and the raw counts were expressed as a log2 fold-change between Non-PAD and CLTI patients.

### 2.7. Quantitative Reverse Transcription Polymerase Chain Reaction (qRT-PCR) 

To confirm the miRNA profile discovery findings obtained using Next Generation Sequencing, we measured the expression of all miRNA which were deregulated by at least 2 folds and *p*-value < 0.05 in CLTI patients relative to non-PAD controls. Specifically, miRNA-1827, miRNA-320e, miRNA-98-3P, miRNA-6843 and miRNA-6766-5P were confirmed and validated in 2 supplementary patient populations. The qRT-PCR was performed through a two-step reaction process: reverse transcription (RT) and PCR (Thermo Fisher, Waltham, MA, USA). miRNA cDNA synthesis was carried out by using a Taqman TM Advanced miRNA cDNA synthesis kit (Thermo Fisher; catalog number: 28007). The miRNAs were quantified using Taqman TM fast advanced master mix (Thermo Fisher; catalog number 4444557) according to the manufacturer’s recommendations. Each reaction was performed in duplicate. A miRNA-484 spike-in control (Qiagen) was selected as the internal control miRNA as the value of miRNA-484 was consistently equal in all samples. The threshold cycle (*C*_t_) values obtained for each investigated miRNA were normalized to the respective control miRNA (miRNA-484) *C*_t_ value to obtain ∆ *C*_t_ values, which were then used to calculate the relative expression and plot the relative fold change values.

### 2.8. Target Prediction, Visualization and Over-Representation Analysis 

Ingenuity Pathway Analysis (IPA) (Qiagen, Venlo, The Netherlands) was used to identify gene targets of miRNA which were validated to be differently expressed in non-PAD controls and CLTI patients. These miRNAs were converged onto genes associated to CLTI. Consensus Path DB (CPDB) (45) was then used to run an over-representation analysis to uncover Gene Ontology (GO) terms and biologic pathways associated to these converging gene targets. 

### 2.9. Statistical Methods 

SPSS software, version 23 (SPSS Inc., Chicago, IL, USA) was used for data entry and analysis. All analyses were carried out at a 5% two-sided significance level. Demographics and baseline measurements were recorded for each patient. Continuous variables were tested for normality using Shapiro-Wilk test and normality plots. Normally distributed continuous variables were summarized and reported in terms of means as well as standard deviation. For non-normally distributed data, the median and interquartile ranges (IQR) were calculated. The categorical variables were reported as percentages. Evaluation of baseline characteristics was done using independent *t*-tests or Mann-Whitney U test for continuous variables. Fisher’s exact test or chi-square test was used for categorical variables. Differences in miRNA levels between the two groups were calculated using non-parametric tests. For the validation phase, we matched cases and controls, in which non-PAD controls were matched to CLTI cases based on age, sex, smoking, hypertension, hyperlipidemia, diabetes and coronary artery disease.

## 3. Results

### 3.1. Cohort Description

A total of 95 subjects were investigated in 3 phases: discovery (*n* = 23), confirmation (*n* = 52) and validation (*n* = 20) phases (Table 1). For each phase, a new cohort of non-PAD controls and CLTI patients were recruited. In the CLTI group of each phase, patients were mainly comprised of males who had a higher prevalence of cardiovascular risk factors as compared to the non-PAD subjects, with the exception of the validation phase due to matching (Table 1). 

### 3.2. Discovery Phase: Global Plasma miRNA Profile of CLTI and Non-PAD Patients Using Next Generation Sequencing 

**Figure 1:**
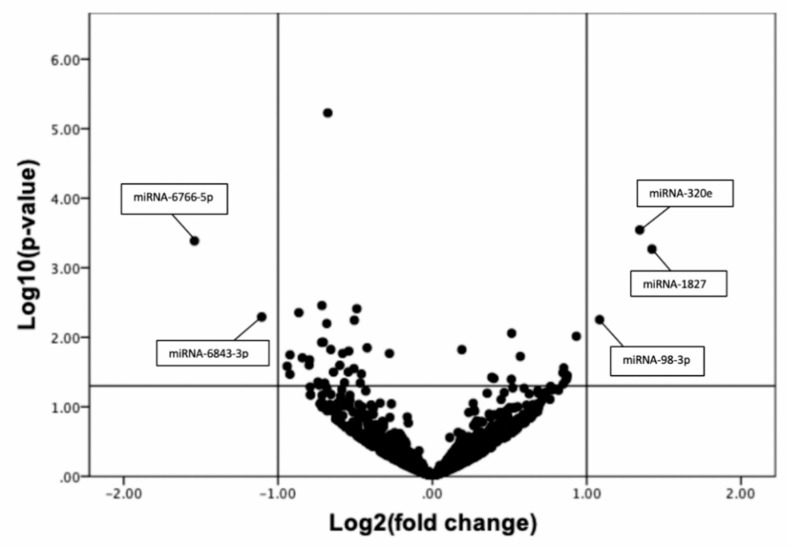
Volcano plot representing the fold change and *p*-values for all miRNA identified via Next Generation Sequencing in CLTI patients (*n* = 13) relative to non-PAD controls (*n* = 10). All miRNA with at least 2-fold increase and *p*-value *p* < 0.05 are shown in the top left box (down regulated) and top right box (up regulated).

A total of 1314 miRNA in plasma were identified by non-targeted next generation sequencing. A volcano plot was constructed to visualize the significant differences between non-PAD and CLTI patients against fold-change (Figure 1). Using two independent parameters (fold change > 2.0 and *p*-value < 0.05), we identified five miRNAs which were significantly deregulated when comparing non-PAD controls and CLTI patients. Specifically, two miRNAs were significantly down regulated (miRNA-6843-3p and miRNA-6766-5p) while three were significantly upregulated (miRNA-1827, miRNA-320 and miRNA-98-3p). Consequently, these significantly deregulated miRNA’s in CLTI group were considered for the confirmation phase. 

### 3.3. Confirmation of Next Generation Sequencing Results by Quantitative qRT-PCR

To confirm the deregulation of the 5 miRNAs identified by next generation sequencing (miRNA-1827, miRNA-320, miRNA-98-3p, miRNA-6843-3p and miRNA-6766-5p), independent qRT-PCR experiments were conducted on a new cohort of and CLTI (*n* = 32) cases and non-PAD (*n* = 20) controls. In terms of demographics and clinical characteristics, significant differences between the two groups pertained to age, ABI values and cardiovascular risk factors (Table 1).

**Figure 2:**
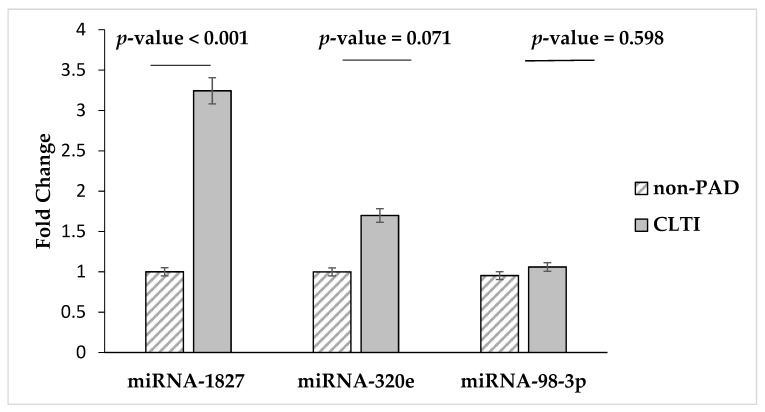
Confirmation of Next Generation Sequencing. The plasma levels of upregulated miRNA in control non-PADs (*n* = 20) and CLTI patients (*n* = 31) were investigated via qRT-PCR. Compared with miRNA levels in control subjects, only miRNA-1827 showed a statistically significant increase in CLTI patients (3.2 folds, *p*-value < 0.001).

The qRT-PCR results confirmed that miRNA-1827 was the only miRNA to be up-regulated in patients with CLTI, with a 3.2-fold increase, compared to non-PAD group (*p*-value < 0.001) (Figure 2). Relative to non-PAD controls, the qRT-PCR results for miRNA-320 and miRNA-98-3p showed increased levels in CLTI patients; however, these findings were not statistically significant. We did not observe any difference in the expression level of miRNA-6843-3p and miRNA-6766-5p. Both of these miRNAs had relatively low CT values in both non-PAD and CLTI patient samples.

### 3.4. Validation of miRNA-1827via qRT-PCR

**Figure 3:**
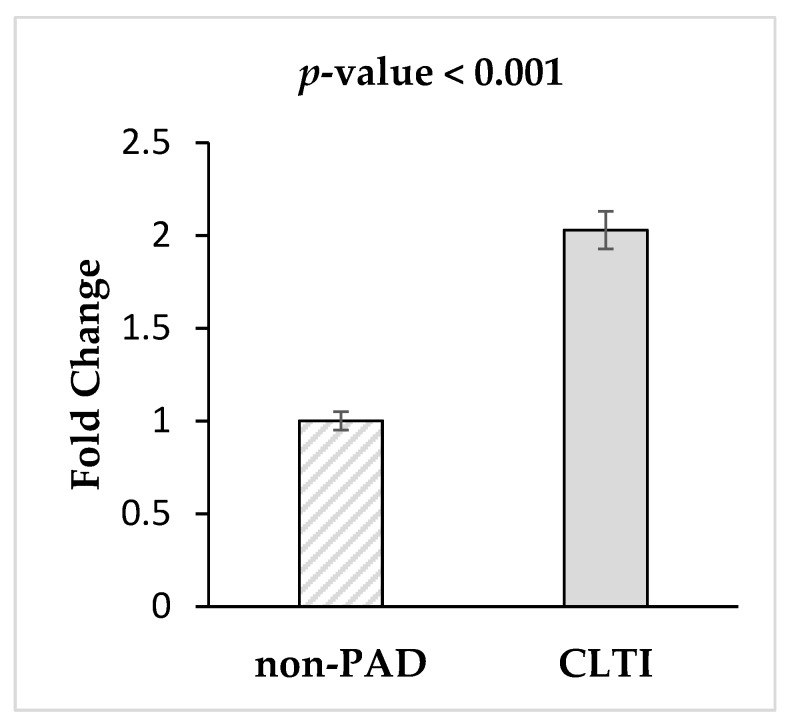
Validation of miRNA-1827 in age-cardiovascular risk factors-matched non-PAD controls (*n* = 10) and CLTI (*n* = 10) patients. miRNA-1827 was validated in a new cohort of patients via qRT-PCR. Compared with miRNA levels in matched control, miRNA-1827 remained significantly increased in CLTI patients (2-folds, *p*-value < 0.001).

To minimize the influence of cardiovascular confounding factors on miRNA-1827 expression in CLTI patients, 10 non-PAD subjects were successfully matched to 10 CLTI patients based on age, sex, smoking, hypertension, hyperlipidemia, diabetes and coronary artery disease, Table 1. Non-parametric test results showed that miRNA-1827 had a significant 2.0-fold increase in the CTLI group relative to non-PAD controls (*p*-value < 0.001) (Figure 3).

### 3.5. miRNA-1827 Target Prediction, Function and Pathway Analysis. 

Using Ingenuity Pathway Analysis (IPA), 22 genes associated to CLTI were found to converge on gene targets of miRNA-1827. Gene Ontology (GO) terms identified as significant (*p* < 0.01) through over-representation analysis were considered. The top five most significant GO terms populated were positive regulation of transport, regulation of protein localization, regulation of transport, response to hormone and negative regulation of protein localization (Table 2). The top five significantly (*p* < 0.01) over-represented pathways were Hydroxycarboxylic acid- binding receptors (Reactome), p53 signaling pathway—homo sapiens human (KEGG), Nanomaterial induced apoptosis (Wikipathways), ceramide signaling pathway (BioCarta) and Apoptosis—multiple species—homo sapiens (KEGG) (Table 3). 

## 4. Discussion

In this pilot study, we sought to identify miRNA associated with CLTI. To achieve this, we used non-targeted next generation sequencing and qRT-PCR to identify and validate deregulated expression of miRNAs in the blood plasma of patients with CLTI compared to non-PAD controls. Our results indicate an increase in circulating miRNA-1827 in patients with CLTI compared to their non-PAD controls. As expected, baseline clinical parameters in our discovery as well as confirmation cohorts revealed higher number of confounding factors in the CLTI group. This is to be expected as cardiovascular risk factors are usually present in PAD patients who progress to CLTI. Therefore, such confounding factors may have an effect on miRNA-1827 expression and contribute to the observed increase in CLTI patients. Therefore, to minimize the effect of confounding factors on miRNA expression, we matched non-PAD to CLTI cases. Despite matching, our data indicates a significant increase in miRNA-1827 levels within the CLTI patient group. Therefore, our results suggest that miRNA-1827 may be a potential biomarker for CLTI; however, future studies with a larger sample size are needed to confirm our findings.

Further investigation into miRNA-1827 using IPA revealed 22 overlapping miRNA sequences that are both targeted by miRNA-1827and previously identified as being involved in severe PAD or CLTI patients. Of those 22 mRNA sequences, the same two proteins, Caspase 8 (CASP8) and beta cell lymphocyte 2 (BCL2), are involved in a number of GO terms and pathways. These primarily include positive regulation of transport and protein localization, p53 signaling, ceramide signaling, and apoptosis. CASP8 is also known to play a central role in executing the activation phase of cell apoptosis [18]. Similarly, the BCL2 family of proteins are known to be key regulators of apoptosis [19]. However, upon conducting Consensus Path DB over-representation analysis, it was revealed that both these proteins play a role in increasing the directed movement rates of macromolecules, small molecules, and ions (into/out of, within, and between cells). miRNA-1827 may potentially attenuate the role of these two proteins in allowing such migration. 

Moreover, the scientific literature is ripe with evidences indicating the immense utility of miRNA-based diagnostics and therapeutic biomarkers [20,21,22]. In the case of CLTI, a biomarker that could identify the early onset of the disease, inform timely treatment options, and subsequently significantly improve patient outcomes, is extremely crucial and urgently needed [23,24]. Fortunately, the significantly altered protein expression of CLTI patients makes the discovery of a biomarker simpler [25]. With regards to the findings from this study, further studies are still needed to confirm the capability of miRNA-1827 to serve as a valid and reliable biomarker of CLTI. However, we believe that this association study was the first step towards that endeavor. 

Other studies have also explored the use of circulating miRNA as potential biomarkers for CLTI but reached vastly different conclusions to the findings from this study. For instance, Li et al. and Cheng et al. have previously identified miRNA-4739 and miRNA-323b-5p as potential biomarkers of CLTI in patients with type-2 diabetes, respectively [26,27]. However, our findings do not demonstrate the overexpression of miRNA-4739 or miRNA-323b-5p in CLTI patients when compared to non-PAD patients. One of the reasons for this disparity in findings could be due to differences in patient cohort selections. Li et al. and Cheng et al. only recruited diabetic patients, whereas we recruited both diabetic and non-diabetic patients. Furthermore, both Li et al. and Cheng et al. used a targeted approach using a microarray that contains human miRNAs to identify their biomarker. In our study, we were the first to utilize a global untargeted approach to identify a circulating miRNA that can serve as a potential biomarker for CLTI. 

Furthermore, many studies have demonstrated that miRNAs play a role in a number of different pathophysiological events pertaining to CLTI, but not in the capacity of biomarkers. For instance, miRNA-150, miRNA-92a, and miRNA-100, among others, have been shown to be regulators of angiogenesis in CLTI models [28,29,30]. Similarly, miRNA-106b-93-25 has been shown to be involved with neovascularization processes that follow ischemia in progenitor/stems cells [31]. miRNA(s) also appear to play a role in other pathophysiological events pertinent to CLTI, such as inflammation, diabetes mellitus, and hypoxia [32,33,34,35,36,37,38]. However, the majority of these findings were discovered in animal models and are yet to be translated in CLTI patients.

With respect to miRNA-1827 itself, a review of the literature revealed a limited number of publications. However, it appears to play a role in tumor suppression [39,40,41]. In contrast, our data indicates an association between miRNA-1827and CLTI. However, we cannot be certain if miRNA-1827 is a cause of CLTI or an effect of having CLTI. Therefore, the exact role that miRNA-1827plays in CLTI is yet to be elucidated. 

The small sample size used in this study was a major limitation. However, this study also has strengths that merit discussion. Firstly, we utilized a non-targeted approach to discovering miRNAs in circulating plasma in all CLTI patients rather than a subgroup of patients with CLTI. Secondly, this study was conducted in a series of 3 phases with a different patient cohort each time. Lastly, our parameters for diagnosis of early CLTI were stringent, providing a more accurate look into our CLTI patient population. 

In summary, our study revealed miRNA-1827 to be significantly up-regulated in CLTI. Our data links miRNA-1827 to CLTI. The exact role each miRNA plays in disease progression was briefly looked into using bioinformatics tools, however further investigations, such as a functional genomic study, are needed to clarify the exact underlying mechanisms these miRNAs are involved in. Lastly, microvesicles and other forms of cardiovascular biomarkers should also be explored as potential diagnostic tools for CLTI.

## Figures and Tables

**Table 1:** General demographics of the study subjects in the discovery, confirmation and validation experiments.

	Discovery Group (*n* = 23)			Confirmation Group (*n* = 52)			Validation Group (*n* = 20)		
	non-PAD (*n* = 10)	CLTI (*n* = 13)	*p*-Value ^α^	non-PAD (*n* = 20)	CLTI (*n* = 32)	*p*-Value ^α^	non-PAD (*n* = 10)	CLTI (*n* = 10)	*p*-Value ^α^
**Mean (SD) †**									
Age	62.4(6.2)	70.3(8.6)	0.02	55.7(15.3)	71.0(7.7)	<0.001	77.9(5.6)	78.6(6.4)	0.733
ABI	1.0 (0.1)	0.4 (0.1)	<0.001	1.1 (0.1)	0.4 (0.1)	<0.001	1.1 (0.1)	0.5 (0.1)	<0.001
**Frequency (%) ‡**									
Sex (male)	5 (50)	9 (69)	0.417	8 (40)	18 (58)	0.258	9 (90)	8 (80)	1.00
Hypertension	5 (50)	10 (77)	0.221	3 (15)	26 (84)	<0.001	8 (80)	9 (90)	1.00
Hypercholesterolemia	5 (50)	12 (92)	0.052	6 (30)	26 (84)	<0.001	5 (50)	9 (90)	0.141
Diabetes	0 (0)	1 (8)	1.00	2 (10)	14 (45)	0.012	3 (30)	2 (20)	1.00
Smoking History	6 (60)	12 (92)	0.127	10 (50)	25 (81)	0.031	5 (50)	8 (80)	0.350
Coronary artery disease	0 (0)	8 (62)	0.003	1 (5)	18 (58)	<0.001	3 (30)	7 (70)	0.179
Stroke	0 (0)	0 (0)	NA	1 (5)	6 (19)	0.229	0 (0)	3 (30)	0.211
**Medication (%) ‡**									
Statin	4 (40)	13 (100)	0.002	6 (30)	24 (77)	0.005	5 (50)	9 (90)	0.141
ACEi/Arb	3 (30)	8 (62)	0.214	3 (15)	20 (65)	0.001	7 (70)	8 (80)	1.00
Beta Blocker	1 (10)	4 (31)	0.339	1 (5)	11 (36)	0.017	3 (30)	7 (70)	0.179
Insulin	0 (0)	0 (0)	NA	0 (0)	4 (13)	0.145	0 (0)	2 (20)	0.474

ABI: Ankle bronchial index, ACEi/Arb: Angiotensin-converting enzyme (ACE) inhibitors. ^α^ the significance of the difference between CLTI and non-PAD groups. † Differences between groups were compared using Mann-Whitney test. ‡ Differences between groups were compared using chi-square test. Means and standard deviations were calculated for continuous variables; all numbers were rounded to one decimal place. Frequencies and percentages were calculated for categorical variables; all numbers were rounded up with zero decimal place. All *p*-values were rounded to three decimal places.

**Table 2:** Top five gene ontologic terms populated by a CPDB over-representation analysis of the miRNA-1827.

GO Term	Overlapping Gene IDs	*p*-Value
Positive regulation of transport	CASP8; HCAR2; AHSG; HCLS1; DAB2; PPID; BCL2	4.34 × 10^−^^5^
Regulation of protein localization	CASP8; HCAR2; HCLS1; DAB2; LCP1; PPID; BCL2	4.80 × 10^−^^5^
Regulation of transport	CASP8; THBS1; HCAR2; AHSG; HCLS1; DAB2; LCP1; PPID; BCL2	5.44 × 10^−^^5^
Response to hormone	BTG2; CASP8; THBS1; AHSG; HCLS1; DAB2; BCL2	5.50 × 10^−^^5^
Negative regulation of metabolic process	TNFSF13; BTG2; THBS1; HCAR2; AHSG; HCLS1; DAB2; PPID; GPRC5A; CTDSP2; BCL2	6.89 × 10^−^^5^

**Table 3:** Top five biologic pathways populated by a CPDB over-representation analysis of miRNA-1827.

Pathway	Protein Members	Source	*p*-Value
Hydroxycarboxylic acid-binding receptors	HCAR3; HCAR2	Reactome	6.07 × 10^−^^6^
P53 signaling pathway—*Homo sapiens* (human)	BCL2; CASP8; THBS1	KEGG	0.0001
Nanomaterial induced apoptosis	CASP8; BCL2	Wikipathways	0.0003
Ceramide signaling pathway	CASP8; BCL2	BioCarta	0.0010
Apoptosis—multiple species—*Homo sapiens* (human)	CASP8; BCL2	KEGG	0.0010
